# Photochemically and Thermally Programmed Optical Multi‐States from a Single Diacetylene‐Functionalized Cyanostilbene Luminogen

**DOI:** 10.1002/advs.202307791

**Published:** 2024-01-15

**Authors:** Jahyeon Koo, Jaeseok Hyeong, Junhwa Jang, Youngjae Wi, Hyeyoon Ko, Minwoo Rim, Seok‐In Lim, Seok‐In Na, Yu‐Jin Choi, Kwang‐Un Jeong

**Affiliations:** ^1^ Department of Polymer‐Nano Science and Technology Department of Nano Convergence Engineering Jeonbuk National University Jeonju 54896 Republic of Korea; ^2^ Department of Flexible and Printable Electronics and LANL‐JBNU Engineering Institute‐Korea Jeonbuk National University Jeonju 54896 Republic of Korea; ^3^ Materials Department University of California Santa Barbara CA 93106 USA

**Keywords:** aggregation‐induced emission, multi‐responsiveness, optical information encryption, photochemical reaction, topochemical polymerization

## Abstract

To develop advanced optical systems, many scientists have endeavored to create smart optical materials which can tune their photophysical properties by changing molecular states. However, optical multi‐states are obtained usually by mixing many dyes or stacking multi‐layered structures. Here, multiple molecular states are tried to be generated with a single dye. In order to achieve the goal, a diacetylene‐functionalized cyanostilbene luminogen (DACSM) is newly synthesized by covalently connecting diacetylene and cyanostilbene molecular functions. Photochemical reaction of cyanostilbene and topochemical polymerization of diacetylene can change the molecular state of DACSM. By thermal stimulations and the photochemical reaction, the conformation of polymerized DACSM is further tuned. The synergetic molecular cooperation of cyanostilbene and diacetylene generates multiple molecular states of DACSM. Utilizing the optical multi‐states achieved from the newly developed DACSM, switchable optical patterns and smart secret codes are successfully demonstrated.

## Graphical abstract

A newly synthesized diacetylene‐functionalized cyanostilbene luminogen (DACSM) exhibits molecular and optical multi‐states due to synergetic molecular cooperation of cyanostilbene and diacetylene functions. By controlling the molecular states with two wavelengths of light and heat, advanced optical applications are successfully demonstrated, such as switchable optical patterns and smart secret codes.

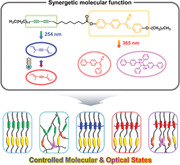

## 1 Introduction

Optical sensing materials enable rapid response to analytes and stimuli with significant variations in color or luminescence. Especially, optical changing systems have been consistently investigated for efficient information delivery and advanced optical applications, such as smart imaging and sensors, optical information encryptions, and multimodal optical systems.^[^ ^1^, ^2^, ^3^, ^4^ ^]^ These systems have sometimes been realized from engineering points, such as stacking different optical elements and fabricating an array.^[^ ^5^, ^6^ ^]^ Based on a diffraction principle, spatially positioning of materials into 2D lattice structure and the lattice modulation has also been achieved for the optical change.^[^ ^7^, ^8^ ^]^ However, the molecular change of dyes under stimuli has played a fundamental key role. Stimuli‐responsive motifs in dyes offer the color‐changing ability by molecular switches including conformational and configurational dynamics under various stimuli, such as heat, light, pressure, pH, electric field, and host‐guest systems.^[^ ^9^, ^10^, ^11^ ^]^ In some cases, combinations of specific stimuli and environmental conditions are required for the desired color change.^[^ ^12^ ^]^ Because the two or more molecular changes on combined conditions occasionally make a new photophysical process.

Advanced multiple‐color generation has recently received considerable attention for encoding multiple and complex information on materials, and advanced visualization systems. It has mainly been achieved using a variety of optical compounds and components.^[^ ^13^, ^14^ ^]^ Furthermore, stimulus‐induced multiple molecular transition behaviors such as electrochemically different states and controlled molecular packing structures have been studied for photophysically multi‐states.^[^ ^15^, ^16^ ^]^ Among the systems reported to date, T. Lin and co‐workers have represented a tetraphenylethylene‐modified cyano‐spiropyran dye exhibiting multiple optical switching behaviors under light, pH, a specific anion, and pressure stimuli.^[^ ^17^ ^]^ S. Zeng et al. reported a set of thermo‐, photo‐, and mechanochromic materials with a multi‐layered structure.^[^ ^18^ ^]^ Despite these advances, there are still challenges facing most applications of distinguishing optical systems with multi‐states and multi‐stimuli responsiveness. Several stimulus‐responsive behaviors such as mechano‐ and chemochromism are often restricted in general optical applications due to their low accessibility or toxicity. In most cases, multi‐component systems and multi‐layered structures were adopted, leading to much more complicated systems with a lot of processes.^[^ ^13^, ^18^, ^19^, ^20^ ^]^ In particular, mixing of different dyes occasionally causes unexpected problems such as phase separation, undesirable photophysical processes, and undefined assembly.^[^ ^20^, ^21^, ^22^ ^]^ Synthesis of desired multi‐tunable organic dyes is an ideal strategy to address these issues, which is still considered a big challenge.^[^ ^17^, ^24^, ^25^, ^26^, ^27^ ^]^

**Scheme 1:**
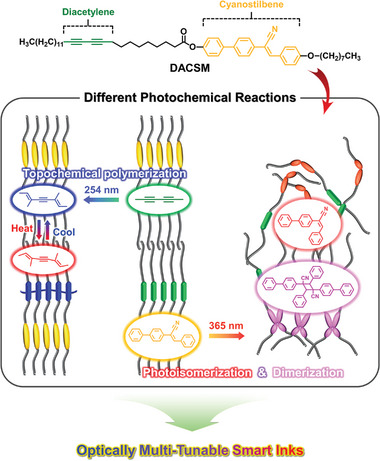
Chemical structure of DACSM and its molecular multi‐states by photo‐ and thermal stimuli.

Here, we newly designed a diacetylene‐functionalized cyanostilbene luminogen (DACSM) in **Scheme** **1** for three reasons. At first, cyanostilbene moiety in DACSM is both strongly emissive in solid state and photochemically reactive under 365 nm light.^[^ ^28^, ^29^ ^]^ Second, polydiacetylene that is formed by topochemical polymerization of self‐assembled diacetylene under 254 nm light shows intrinsic thermochromic behavior by perturbations and conformational rotation of the polydiacetylene backbone.^[^ ^30^, ^31^, ^32^ ^]^ Finally, in view of molecular geometry, we expected a self‐assembled structure that is effective in eliciting synergetic molecular functions of two moieties. In response to our expectations, the photochemical reactions of two moieties in DACSM result in multi‐color and luminescent states, with the benefits of light processing. Each molecular and optical change of DACSM under photo‐ and thermal stimuli and their relationships were precisely identified. Based on the tunable optical properties of DACSM, we further demonstrated smart optical patterns that could play a pivotal role in advanced information encryption and anti‐counterfeiting.

## 2 Results and Discussion

### 2.1 Self‐Assembly Behavior and Structure Identification of DACSM

To derive the synergetic optical properties of diacetylene and cyanostilbene moieties from a single molecule, a cyanostilbene‐based liquid crystalline (LC) molecule with alkyl chain (compound 3 in Figure S1, Supporting Information) was chemically connected with pentacosa‐10,12‐diynoic acid by esterification. The detailed synthetic procedure of targeted diacetylene‐functionalized cyanostilbene luminogen (DACSM) is described in Figure S1 (Supporting Information). The chemical structure and purity of the intermediates and DACSM were confirmed by proton (^1^H) and carbon (^13^C) nuclear magnetic resonance (NMR) analyses, gas chromatography (GC/MS), and matrix‐assisted laser desorption/ionization time of flight (MALDI‐ToF) mass spectroscopy (Figures S2–S10, Supporting Information). The detected molecular weight of DACSM was 804.46 g mol^−1^, corresponding to the sum of the theoretical molecular weight and sodium ion.

**Figure 1:**
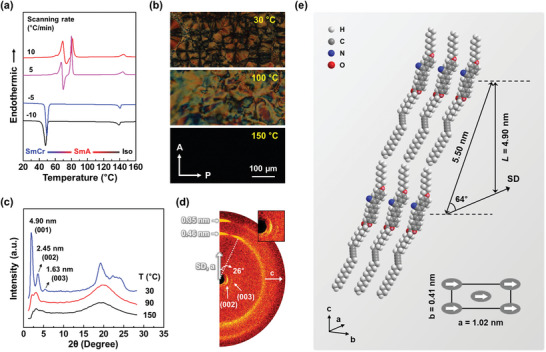
Self‐assembly behavior of DACSM: a) DSC thermogram of DACSM. b) POM textures of DACSM upon cooling process from 150 to 30 °C at 5°C min^−1^. c) 1D WAXD patterns of DACSM at different temperatures. d) 2D WAXD pattern of the shear‐induced oriented DACSM. e) Molecular packing model of the self‐assembled DACSM.

The self‐assembled behavior of DACSM was first investigated by differential scanning calorimetry (DSC) and polarized optical microscopy (POM) analyses at different temperatures. In the DSC thermograms, two exothermic peaks are detected during cooling cycles (**Figure** **1**a). A small peak at 140 °C corresponds to the phase transition from isotropic (Iso) state to LC mesophase. The other large peak at 48 °C indicates the crystallization of DACSM from its LC mesophase. On heating cycles, the melting and recrystallization of the crystal occur at ≈70 °C. A following endothermic peak at 80 °C indicates the transition to LC mesophase. The isotropization of DACSM is detected at a higher temperature, which is almost identical to the temperature of Iso‐to‐LC phase transition upon cooling. Considering a large portion of flexible alkyl chains in DACSM, a relatively high isotropization temperature (*T*_i_ = 140 °C) may be affected by a strong lateral molecular interaction between cyanostilbene moieties. The DSC thermal trace of DACSM is not significantly changed at 5 and 10 °C min^−1^. To evaluate the formation of the ordered structure, thermal transition behavior of DACSM was further monitored by POM observations on cooling process from 150 to 30 °C at 5 °C min^−1^ (Figure 1b). When the temperature decreases below 140 °C, a dark image indicating Iso state is changed to a Schlieren texture. Crystallized multi‐domains are observed upon further cooling to 30 °C. The POM results combined with those of DSC indicate that two ordered phases are exhibited below *T*_i_: a LC phase and a crystal phase.

To identify the thermal phase transitions of DACSM in detail, 1D wide‐angle X‐ray diffraction (WAXD) analysis as a function of temperatures was conducted (Figure 1c). Typical amorphous halos indicating the short positional order of DACSM molecules are observed above *T*_i_. As the temperature goes down below *T*_i_, a diffraction peak at 2*θ* = 3.18° newly comes out. The corresponding *d*‐spacing (*d* = 2.77 nm) is approximately matched with the half value of an optimized DACSM molecular length (*l* = 5.50 nm) (Figure S11, Supporting Information). In addition, the scattering halo in the high angle region (2*θ* = 15° ∼ 25°, *d* = 0.45 nm) becomes a little stronger and sharper due to the lateral molecular packing between DACSM molecules. The additional temperature decline to 30°C leads to many sharp diffraction peaks, which means that DACSM solidifies through crystallization. The 2*θ* value ratio of three diffractions at *d* = 4.90, 2.45, and 1.63 nm in the low angle is 1:2:3, indicating a layered structure with a layer spacing (*L*) of 4.90 nm.^[^ ^33^, ^34^ ^]^ In the high‐angle region, sharp diffractions including a prominent peak at 19.23° (*d* = 0.46 nm) are overlapped. As a result, it is realized that DACSM sequentially exhibits Iso, smectic A (SmA), and smectic crystal (SmCr) phases upon cooling (Iso‐SmA‐SmCr).

To investigate the molecular packing structure of the crystalline DACSM, 2D WAXD patterns of the mechanically oriented DACSM were obtained by radiating X‐ray normal to shear direction (SD) (Figure 1d). Two diffractions on the equator (assigned as (002) and (003)) correspond to the high‐order diffractions of the first‐order one (*L* = 4.90 nm). On the quadrants, diffraction arcs at 2*θ* = 19.49° (*d* = 0.45 nm) are 24° away from the meridian, indicating that the layer building block is tilted ≈24° from the layer normal.^[^ ^33^, ^34^ ^]^ The other diffractions at 2*θ* = 19.35° and 25.07° (*d* = 0.46 and 0.35 nm) on the meridian are directly related to the lateral molecular packing with long‐range positional order, and are assigned to (200) and (110), respectively.^[^ ^33^, ^34^ ^]^ The careful refinement of the reciprocal lattice based on the 2D WAXD patterns suggests that the crystalline DACSM (SmCr phase) has a monoclinic unit cell with the dimensions of *a* = 1.02 nm, *b* = 0.41 nm, *c* = 5.50 nm, *α* = *γ* = 90°, and *β* = 64°. The corresponding molecular packing model is illustrated in Figure 1e.

### 2.2 Optical Changes of DACSM by Photochemical Reactions of Cyanostilbene Moiety

**Figure 2:**
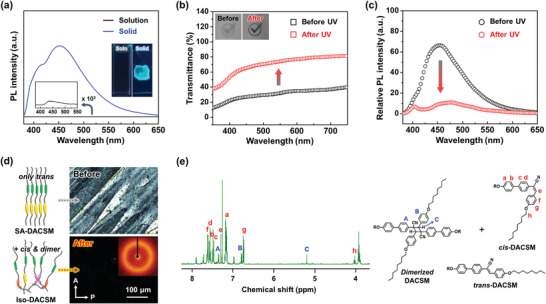
Photochemically reactive DACSM under 365 nm light: a) PL spectra of DACSM in solution and solid states (inset: corresponding photographs). b) Transmittance, c) PL spectra, and d) POM texture of SA‐DACSM film upon 365 nm light irradiation (inset: 2D WAXD pattern of Iso‐DACSM). e) ^1^H NMR spectrum of Iso‐DACSM formed by UV light.

Photophysical properties of DACSM were investigated by ultraviolet‐visible (UV–vis) and photoluminescence (PL) spectroscopy. DACSM dissolved in tetrahydrofuran (THF) at 60 µM concentration shows an absorption band at *λ*_max_ = 353 nm (Figure S12, Supporting Information). The solution exhibits an extremely low PL intensity at *λ*_max_ = 436 nm which is difficult to detect with the naked eye (**Figure** **2**a). It is due to a twisted intramolecular charge transfer (TICT) of cyanostilbene moiety in DACSM.^[^ ^35^, ^36^ ^]^ However, the drop‐casted and self‐assembled DACSM (SA‐DACSM) is much more strongly emissive at *λ*_max_ = 453 nm. The definite contrast in PL intensity between solution and solid states is shown in the inset of Figure 2a, representing the aggregation‐induced emission (AIE) effect of the cyanostilbene moiety.^[^ ^35^, ^37^ ^]^ To confirm photochemical reactivity of DACSM, the DACSM solution was irradiated by 365 nm light with 20 mW cm^−2^ intensity (Figure S13a, Supporting Information). The irradiated solution for 5 min exhibits a blue‐shifted absorption band (*λ*_max_ = 353 nm to *λ*_max_ = 250 nm) in Figure S13b (Supporting Information), which originates from the photoisomerization from *trans*‐ to *cis*‐DACSM with a shorter effective conjugated length.^[^ ^35^, ^36^, ^37^ ^]^ Furthermore, a little restricted TICT of *cis*‐DACSM induces a relatively enhanced emission band at 410 nm (Figure S13c, Supporting Information).^[^ ^35^, ^36^ ^]^ The emergence of *cis*‐DACSM upon UV irradiation is also evidenced by ^1^H NMR analysis in Figure S14 (Supporting Information).^[^ ^35^, ^37^ ^]^

Having established the photophysical/chemical behaviors of DACSM solution, we analyzed DACSM thin films to observe optical spectral changes of SA‐DACSM upon 365 nm light irradiation. After the irradiation of 365 nm light (UV intensity: 20 mW cm^−2^) for 15 min, the translucent film becomes a much transparent film (Figure 2b). Background check mark is shown more clearly as the transmittance at 550 nm increases from 30% to 75% (inset of Figure 2b). The transmittance change is a consequence of phase transition from SmCr to Iso state upon UV irradiation. As shown in Figure 2d, the irradiated SA‐DACSM with 365 nm light (Iso‐DACSM) exhibits a dark image indicating Iso when observed by POM. The inset 2D WAXD pattern in Figure 2d also supports the disordered state of Iso‐DACSM. Due to the photochemical transformation of *trans*‐DACSM to *cis*‐DACSM and dimerized species, and the following disassembly, the emission is quenched according to the AIE principle, where its maximum PL wavelength is found to be 480 nm due to the presence of *cis*‐ and dimerized DACSM (Figure 2c). In addition to the previously discussed *cis*‐DACSM, the emergence of dimerized DACSM species by cycloaddition reaction after 365 nm light irradiation was identified by ^1^H NMR analysis (Figure 2e). The detected proton at 5.21 ppm indicates cyclobutane ring in the dimerized DACSM.^[^ ^37^, ^38^ ^]^ Therefore, Iso‐DACSM contains *trans*‐ and *cis*‐DACSM, and dimerized DACSM. By quantifying their integral values in the ^1^H NMR spectrum, the content of *trans*‐ and *cis*‐DACSM, and dimerized DACSM was calculated to be 36%, 45%, and 19%, respectively. From these experimental results, we concluded that photochemical reactions of cyanostilbene moiety in DACSM under 365 nm light induce different chemical species and disassembly, resulting in changes in both transmittance and luminescence.

### 2.3 Topochemically Polymerized DACSM and its Changes by Thermal and Photo Stimulations

**Figure 3:**
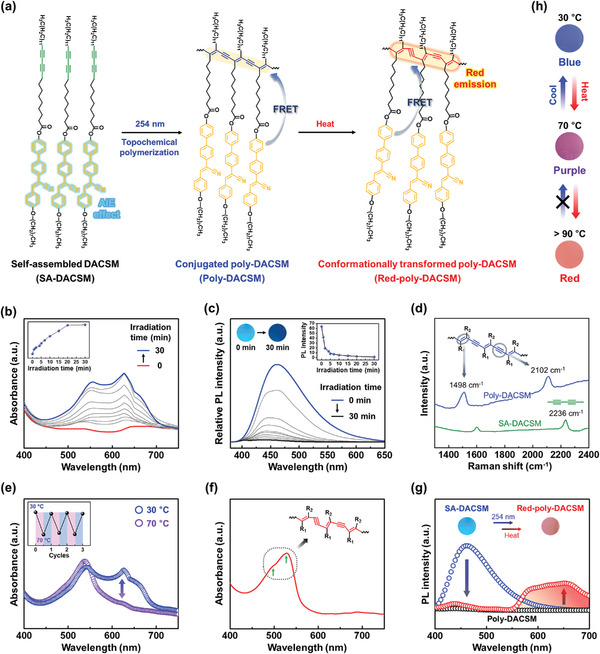
Topochemically polymerized DACSM and its temperature‐dependent changes: a) Schematic illustration for topochemical polymerization of SA‐DACSM under 254 nm light and conformational transformation of the polymerized one by heat. b) UV–vis and c) PL spectra of SA‐DACSM during 254 nm light irradiation (inset: maximum absorption and emission intensity plot with different irradiation time). d) Raman spectrum of SA‐ and Poly‐DACSM. e) UV–vis spectra of Poly‐DACSM at 30 and 70 °C (inset: absorption intensity at 628 nm on a repeatable temperature change between 30 and 70 °C). f) UV–vis of Red‐poly‐DACSM. g) PL spectra of SA‐, Poly‐, and Red‐poly‐DACSM (inset: photographs for emissive SA‐ and Red‐poly‐DACSM under 365 nm). h) Temperature‐dependent color change behavior of Poly‐DACSM.

SA‐DACSM can also be topochemically polymerized under 254 nm due to its close lateral molecular packing structure, which can lead to different photophysical properties including both absorption and luminescence (**Figure** **3**a). By polymerization of the diacetylene group under 254 nm light, a broad absorbance over a visible region (*λ*_max_ = 628 nm) is gradually increased (Figure 3b) and the emission band at *λ*_max_ = 462 nm is dramatically quenched (Figure 3c). The formation of polydiacetylene upon 254 nm light irradiation is further identified by the Raman spectrum (Figure 3d). The absorption peaks at 1498 and 2102 cm^−1^ correspond to straightly conjugated alkene‐alkyne groups in the polydiacetylene. However, this chemical change does not make notable variation in molecular packing structure (Figure S15, Supporting Information).^[^ ^39^, ^40^ ^]^ As a result, topochemically polymerized DACSM (Poly‐DACSM) has almost identical molecular packing structure with SA‐DACSM and exhibits a blue color and an extremely quenched emission due to the absorption of polydiacetylene at *λ*_max_ = 628 nm and Förster resonance energy transfer (FRET) effect from cyanostilbene to polydiacetylene, respectively.^[^ ^41^, ^42^, ^43^ ^]^

Additionally, Poly‐DACSM exhibits a thermochromic behavior due to the presence of polydiacetylene. When Poly‐DACSM is heated to 70 °C, the absorption at 628 nm decreases while the absorption at 537 nm increases (Figure 3e). It indicates that the effective conjugation length of polydiacetylene is shortened. The absorption band returns to its initial state when cooling to 30 °C. According to a repeatable temperature change between 30 and 70°C, the color of Poly‐DACSM is reversibly switched between blue and purple (inset graph of Figure 3e). A higher thermal energy input above 80°C causes an irreversible color change to a red color. After a thermal treatment (heating to 100 °C and then cooling to room temperature), the absorption at 628 nm is completely extinguished (Figure 3f). On the other hand, the absorptions at 500 and 529 nm corresponding to conformationally transformed polydiacetylene with short effective conjugation lengths are enhanced. As shown in Figure S16 (Supporting Information), the measured Raman spectrum after the thermal treatment exhibits red‐shifted peaks at 1517 and 2120 cm^−1^, indicating the conformationally transformed Poly‐DACSM by heat (denoted as Red‐poly‐DACSM).^[^ ^30^, ^44^, ^45^, ^46^, ^47^, ^48^ ^]^ The temperature dependent color change behavior of Poly‐DACSM is related to its phase transition behavior. As shown in Figure S17a (Supporting Information), a morphological texture of Poly‐DACSM thin film does not change within 70 °C. A little color change is only represented by its absorption change. When Poly‐DACSM is cooled, the POM texture returns back to the initial state. The increased molecular motions of alkyl chains and polydiacetylene backbones at that temperature cause a little conformational fluctuation of polydiacetylene, resulting in the color switching between blue and purple.^[^ ^44^, ^45^, ^46^, ^47^, ^48^ ^]^ On the other hand, a higher temperature above 80 °C leads to phase transition from crystalline to LC mesophase. Mobile LC domains are observed at 100 °C (Figure S17b, Supporting Information). The movement of cyanostilbene moities upon their lateral molecular packing breakdown and highly mobile chains result in conformational transformation of polydiacetylene. The conformationally transformed polydiacetylene does not come back to the initial state when cooling to room temperature. This result is due to the imperfect crystallization of the conjugated polymer (Poly‐DACSM) which was supported by POM and DSC experiments between 30 and 100 °C. The POM texture after cooling shows a lower birefringence than the texture before heating, as shown in Figure S17b (Supporting Information). Enthapy changes for disassembly and assembly of Poly‐DACSM upon the thermal treatment are calculated to be 57.4 and 49.2 J g^−1^, respectively (Figure S17c, Supporting Information). Complete conformational transformation of Poly‐DACSM induced by severe molecular motions at a high temperature results in poor molecular ordering when cooling.^[^ ^44^, ^45^, ^46^, ^47^ ^]^ The different color change behavior of Poly‐DACSM with different thermal energy inputs is shown in Figure 3h. Furthermore, the absence of absorption at 628 nm originated from the conformational transformation of Poly‐DACSM leads to a reddish emission by FRET effect.^[^ ^41^, ^43^ ^]^ The emission band of cyanostilbene moiety (400–550 nm) overlaps with the absorption band of the conformationally transformed diacetylene (450–550 nm). As shown in Figure 3g, Red‐poly‐DACSM exhibits a differentiated emission band at *λ*_max_ = 651 nm, compared to that of SA‐ and Poly‐DACSM.

**Figure 4:**
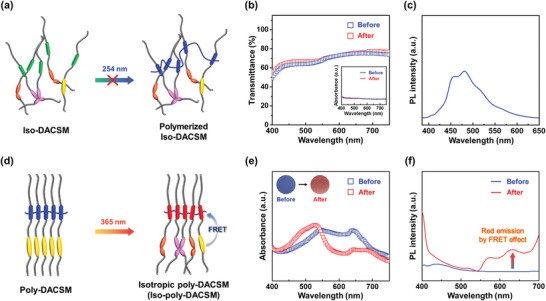
Photochemically reactive Poly‐DACSM under 365 nm light: a) Schematic illustration for the molecular state of Iso‐DACSM upon 254 nm light irradiation. b) Transmittance of Iso‐DACSM before and after 254 nm light irradiation (inset: corresponding absorption spectra). c) PL spectrum of Iso‐DACSM after 254 nm light irradiation. d) Schematic illustration for the molecular state of Poly‐DACSM upon 365 nm light irradiation. e) UV–vis spectra (inset: corresponding photographs) and f) PL spectra of Poly‐DACSM before and after 365 nm light irradiation.

Optical changes upon consecutive light irradiations with two different wavelengths of 254 and 365 nm were further observed. The Iso‐DACSM obtained by 365 nm light irradiation is additionally irradiated with 254 nm light, resulting in no significant change in optical properties (**Figure** **4**a). The almost same absorption and transmittance spectra were obtained after 254 nm light irradiation (Figure 4b). As shown in Figure 4c, the PL spectrum after the irradiation is also identical to the PL spectrum of Iso‐DACSM previously shown in Figure 2c. It is not a surprise that diacetylene moieties in Iso‐DACSM cannot be polymerized under 254 nm light due to the dislocation and long distance between them.^[^ ^49^, ^50^ ^]^ On the other hand, when Poly‐DACSM is irradiated with 365 nm light, significant optical changes are detected from blue to red (Figure 4d). The 365 nm light irradiated Poly‐DACSM (Iso‐poly‐DACSM) exhibits a higher transmittance and absorption at *λ* = 529 nm corresponding to the conformationally transformed polydiacetylene (Figure 4e). In common with Red‐poly‐DACSM, Iso‐poly‐DACSM shows a red emission by the FRET effect (Figure 4f). The photochemical transformation of cyanostilbene under 365 nm light induces phase transition to Iso state and the conformational change of polydiacetylene. The Iso state induced by photochemical reactions of cyanostilbene is proved by amorphous halos in 1D WAXD pattern of Iso‐poly‐DACSM (Figure S18, Supporting Information). The conformationally transformed polydiacetylene in Iso‐poly‐DACSM is also confirmed by Raman shifts at 1517 and 2120 cm^−1^ (Figure S19, Supporting Information). From these results, it is realized that Poly‐DACSM is photochemically reactive and the resultant Iso‐poly‐DACSM exhibits reddish color and emission.

### 2.4 Demonstrations Based on the Programmed Multiple Molecular States of DACSM

**Figure 5:**
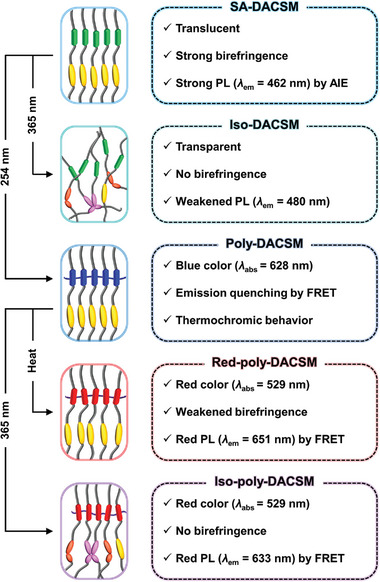
Five different molecular states of DACSM by photo‐ and thermal‐ stimuli and their optical features.

**Figure 6:**
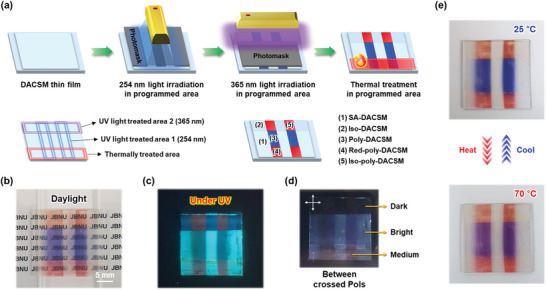
a) Schematic illustration for the fabrication process of a patterned optical film from DACSM. The prepared optical film under b) daylight and c) 365 nm UV light. d) Photograph of the optical film between crossed polarizers. e) Thermochromic behavior of the optical film between 25 and 70 °C.

Molecular and optical changes of SA‐DACSM induced by two wavelengths of light (254 and 365 nm) and thermal treatment (heating to 100 °C and cooling to room temperature) are briefly summarized in **Figure** **5**. For a better understanding, luminescence color information, PL quantum yield (PLQY), and lifetime of each state are additionally provided in Figures S20–S22 (Supporting Information). The PL characteristics of each states are summarized in **Table** **1**. These behaviors of DACSM can be applied in fabricating smart optical patterns. As shown in **Figure** **6**a, a patterned optical film was fabricated by a continuous process of 254 and 365 nm light irradiations and a thermal treatment in programmed areas. From the process, we could encode five different molecular states in each area (assigned as (1)–(5)) on thin film. The corresponding optical contrast is clearly shown under daylight (Figure 6b) and UV light (Figure 6c). When the patterned optical film is located between crossed polarizers, another optical sign emerges due to the different molecular packing states and the corresponding birefringence intensity (Figure 6d; Figure S23, Supporting Information). Unlike the other areas, the 365 nm light‐treated area is completely dark state because of disordered molecular state. The catch here is that (4) and (5) areas exhibit an almost identical absorption and emission color, but they are distinguished by birefringence intensity. Furthermore, the (3) area corresponding to Poly‐DACSM shows a repeatable color switch between blue and purple within the temperature range (≈80 °C) (Figure 6e).

**Table 1:** PL characteristics of five different DACSM states.

Molecular state	𝜆_ex_ [nm]^a)^	𝜆_em_ [nm]^b)^	Φ_PL_ [%]^c)^	τ_1_ [ns]^d)^
SA‐DACSM	374	462	10.1	2.94
Iso‐DACSM	374	480	2.4	1.08
Poly‐DACSM	374	436	0.3	1.01
Red‐poly‐DACSM	374	651	1.1	0.70
Iso‐poly‐DACSM	374	633	0.9	0.84

^a)^ excitation wavelength;

^b)^ emission wavelength;

^c)^ absolute PLQY;

^d)^ PL lifetime.

**Figure 7:**
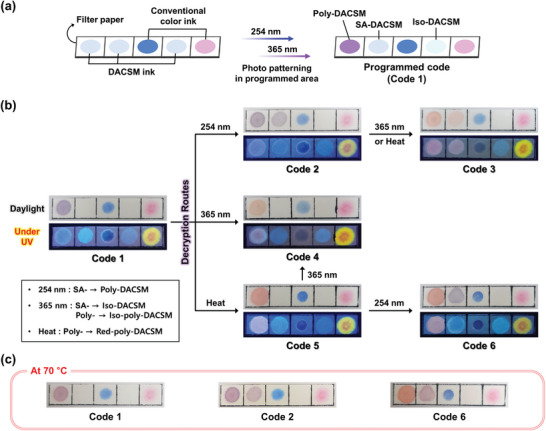
a) Schematic illustration for the fabrication process of a programmed secret code using DACSM and conventional inks. b) The variable secret code depending on decryption routes. c) Photographs of code 1, 2, and 6 at 70 °C.

Utilizing a diversity of optical properties of DACSM, we demonstrated smart secret codes. A precursive code was prepared by painting the DACSM ink, and conventional blue and pink inks on filter paper, followed by photopatterning with 254 and 365 nm lights (**Figure** **7**a). The programmed code (Code 1) can be transformed into other codes (Code 2–6), through different routes (two wavelengths of light and heat) in Figure 7b. The 254 nm irradiation changes SA‐DACSM into Poly‐DACSM. By irradiating 365 nm light, SA‐ and Poly‐DACSM are transformed into Iso‐ and Iso‐poly‐DACSM, respectively. The thermal treatment turns Poly‐DACSM into Red‐poly‐DACSM. Furthermore, the codes containing Poly‐DACSM have a thermoresponsive function owing to the thermochromism of Poly‐DACSM (Figure 7c). These optical performances of the demonstrated secret code can provide meaningful insights for optical information encryption and anti‐counterfeiting.

## 3 Conclusion

To maximize the synergetic optical effects of cyanostilbene and diacetylene moieties, a diacetylene‐functionalized cyanostilbene luminogen (DACSM) was newly designed and synthesized. DACSM exhibits not only aggregation‐induced emission at 462 nm, but also photo‐induced phase transition with optical changes, attributed to the photochemical reactions of cyanostilbene moiety under 365 nm light. The emerged photochemical products such as *cis*‐ and dimerized DACSM induce a disordered state that is transparent and emissive at 480 nm. Furthermore, its close lateral molecular packing allows the self‐assembled DACSM to be topochemically polymerized under 254 nm light. The polymerized DACSM (Poly‐DACSM) has a broad absorption band at 628 nm, and is non‐emissive by Förster resonance energy transfer (FRET) from cyanostilbene to polydiacetylene. Poly‐DACSM can be conformationally transformed by high thermal stimulation and the photochemical reactions of cyanostilbene moiety. The transformed molecular states show red color and emission owing to the shortened effective conjugation length and FRET effect. In addition, Poly‐DACSM exhibits a temperature‐dependent color change (reversible switch between blue and purple below 80°C and irreversible change to red color above 80 °C), which is related to the molecular motion and disassembly behavior of Poly‐DACSM. With these synergetic optical properties of DACSM depending on molecular states, we successfully demonstrated advanced optical platforms, which can be applied to multi‐tunable smart inks and high‐level optical information encryptions.

## Conflict of Interest

The authors declare no conflict of interest.

## Supporting information

Supporting Information

## Data Availability

The data that support the findings of this study are available from the corresponding author upon reasonable request.
