# Supplementary material for: Photochemically and Thermally Programmed Optical Multi‐States from a Single Diacetylene‐Functionalized Cyanostilbene Luminogen
Source: Adv Sci (Weinh). 2024 Jan 15;11(11):2307791. doi: 10.1002/advs.202307791 (PMC10953535; doi:10.1002/advs.202307791)
Supplement: Supplementary file 1 — Supporting Information [file ADVS-11-2307791-s001.pdf]

ADVANCED SCIENCE
Open Access

Supporting Information

for *Adv. Sci.*, DOI 10.1002/advs.202307791

Photochemically and Thermally Programmed Optical Multi-States from a Single Diacetylene-Functionalized Cyanostilbene Luminogen

Jahyeon Koo, Jaeseok Hyeong, Junhwa Jang, Youngjae Wi, Hyeyoon Ko, Minwoo Rim, Seok-In Lim, Seok-In Na, Yu-Jin Choi and Kwang-Un Jeong**

Supporting Information

Photochemically and Thermally Programmed Optical Multi-States from A Single Diacetylene-Functionalized Cyanostilbene Luminogen

Jahyeon Koo, Jaeseok Hyeong, Junhwa Jang, Youngjae Wi, Hyeyoon Ko, Minwoo Rim, Seok-In Lim, Seok-In Na, Yu-Jin Choi,* and Kwang-Un Jeong*

J. Koo, J. Hyeong, J. Jang, Y. Wi, H. Ko, M. Rim, S.-I. Lim, Prof. K.-U. Jeong
Department of Polymer-Nano Science and Technology, Department of Nano Convergence Engineering
Jeonbuk National University
Jeonju 54896, Republic of Korea
E-mail: kujeong@jbnu.ac.kr

Prof. S.-I. Na
Department of Flexible and Printable Electronics and LANL-JBNU Engineering Institute-Korea
Jeonbuk National University
Jeonju 54896, Republic of Korea

Y.-J. Choi
Materials Department
University of California, Santa Barbara
CA 93106, USA
E-mail: yujinc@ucsb.edu

Materials

4-Bromobenzyl cyanide (98%, TCI), 4-hydroxyphenylboronic acid (TCI), 4-hydroxybenzaldehyde (97%, Merck), 1-bromooctane (98%, TCI), pentacosa-10, 12-diynoic acid (97%, Merck), tetrakis(triphenylphosphine)-palladium(0) (99%, Merck), sodium carbonate (Na_2CO_3, 99%, Showa), potassium carbonate (K_2CO_3, 99.5%, Showa), sodium sulfate (Na_2SO_4, 99%, Showa), sodium hydroxide (NaOH, 93%, Showa), hydrochloric acid (HCl, 37%, Merck), tetrahydrofuran (THF, 99.5%, Showa), N,N-dimethylformamide (DMF, 99.5%, Showa), ethyl acetate (EA, 99.5%, Samchun Chemical), n-hexane (95%, Samchun Chemical), ethanol (EtOH, 99%, Merck), dichloromethane (DCM, 99%, Showa), silica gel (63-200, Merck) were used as received.

Synthesis

2-(4'-hydroxy-[1,1'-biphenyl]-4-yl)acetonitrile (1): 4-Bromobenzyl cyanide (2.0 g, 10.20 mmol), 4-hydroxyphenylboronic acid (2.0 g, 14.50 mmol), and $Pd(PPh_3)_4$ (0.1 g, 0.09 mmol) in THF (50 mL) were stirred and heated to 75 °C. Subsequently, a solution of Na_2CO_3 (3.0 g, 21.71 mmol) in water (15 mL) was added into the reaction mixture. The mixture was stirred for 24 h under nitrogen atmosphere. The reaction mixture was cooled at room temperature, after which EA (50 mL) was added. The mixture was washed with distilled water three times and separated by separatory funnel. The obtained organic phase was dried over Na_2SO_4. The crude product was purified by column chromatography with silica gel using n-Hexane:EA = 3:2 to afford compound 1 as ivory solid. (Yield: 49%, 1.04 g). ^{1}H NMR (500 MHz, $CDCl_3$): δ = 7.55 (d, 2H), 7.46 (d, 2H), 7.37 (d, 2H), 6.92 (d, 2H), 4.83 (s, 1H), 3.78 (s, 2H). GC/MS: 183.62 [M--C≡N], 210.62.

4-(octyloxy)benzaldehyde (2): 4-Hydroxybenzaldehyde (2.0 g, 16.37mmol), 1-bromooctane (4.0 g, 20.71 mmol) and K_2CO_3 (3.0 g, 21.71 mmol) were stirred in DMF (40 mL). The mixture was heated at 75 °C and then stirred for 12 h. The reaction mixture was cooled to room temperature and poured into 200 mL of distilled water. The mixture was extracted with EA (50 mL × 3 times) and the combined organic phase was subsequently dried over Na_2SO_4. The crude product was purified by column chromatography with silica gel using n-hexane:EA = 5:1 to afford compound 2 (Yield: 69%, 2.63 g). ^{1}H NMR (500 MHz, $CDCl_3$): δ = 9.88 (s, 1H), 7.82 (d, 2H), 6.98 (d, 2H), 4.03 (t, 2H), 1.81 (m, 2H), 1.46 (m, 2H), 1.23-1.40 (m, 8H), 0.88 (t, 3H). GC/MS: 235.73.

(Z)-2-(4'-hydroxy-[1,1'-biphenyl]-4-yl)-3-(4-(octyloxy)phenyl)acrylonitrile (3): Compound 1 (1.0 g, 4.78 mmol) and compound 2 (1.2 g, 5.12 mmol) were dissolved in EtOH (30 mL), after which NaOH (0.8 g, 20 mmol) was added. The reaction mixture was stirred at room temperature for 24 h. The mixture was neutralized with HCl and then the precipitate was filtered. The precipitate was washed with EtOH and water solution several times to afford compound 3 as yellowish solid (Yield: 72%, 1.46 g). ^{1}H NMR (500 MHz, $CDCl_3$): δ = 7.88 (d, 2H), 7.71 (d, 2H), 7.60 (d, 2H), 7.52 (d, 2H), 7.49 (s, 1H), 6.95 (m, 4H), 4.91 (s, 1H), 4.03 (t, 2H), 1.81 (m, 2H), 1.47 (m, 2H), 1.23-1.41 (m, 8H), 0.89 (t, 3H). GC/MS: 426.55.

DACSM: Compound 3 (0.2 g, 0.47 mmol), pentacosa-10, 12-diynoic acid (0.25 g, 0.67 mmol), EDC (0.5 g, 3.22 mmol), and DMAP (0.05 g, 0.41 mmol) were dissolved in DCM (15 mL). The reaction mixture was stirred at room temperature for 24 h. The mixture was washed with distilled water three times and separated by a separatory funnel. The separated organic phase was dried over Na_2SO_4. The crude product was purified by column chromatography with silica gel using n-Hexane:EA = 9:1 to afford DACSM as yellowish solid. (Yield: 52%, 0.19 g). ^{1}H NMR (500 MHz, $CDCl_3$): δ = 7.88 (d, 2H), 7.71 (d, 2H), 7.59-7.65 (m, 4H), 7.49

(s, 1H), 7.18 (d, 2H), 6.97 (d, 2H), 4.02 (t, 2H), 2.58 (t, 4H), 2.24 (t, 2H), 1.72-1.85 (m, 4H), 1.42-1.58 (m, 6H), 1.22-1.42 (m, 34H), 0.89 (t, 3H).; ^{13}C NMR (125 MHz, $CDCl_3$): δ = 172.4, 161.1, 150.5, 141.8, 140.7, 137.8, 133.9, 131.1, 128.2, 127.7, 126.3, 126.1, 122.1, 118.6, 115.0, 114.8, 107.9, 77.2, 68.2, 65.1, 34.3, 32.0, 28.2-30.0, 26.0, 22.8, 19.2, 18.5, 14.1.; MS(MALDI-ToF) m/z calcd: 804.53 $[M+Na]^+$, found: 804.46.

Specific Notes

For smooth photochemical reactions of DACSM in solid state, DACSM thin films were prepared by shear coating at 60 ~ 70 °C using a bar coater (Elcometer 3530/2) with 5μm gap. The 365 nm light irradiation (intensity: 20 mW/cm^2) for photochemical reactions of cyanostilbene moiety is carried out for 15 min. Topochemical polymerization of DACSM is performed under 254 nm light (intensity: ~1 mW/cm^2) for 30 min.

Characterization

The chemical structure and purity of intermediates and DACSM were confirmed by nuclear magnetic resonance (NMR, JEOL, JNM-EX400) in deuterated chloroform. Chemical shifts were quoted in parts per million (ppm) with tetramethylsilane (TMS) used as a reference. NMR spectroscopy was also utilized to analyze the photoisomerization and cycloaddition of DACSM. Gas chromatography mass spectroscopy (GC/MS) was used to confirm the chemical structure of DACSM intermediates. Matrix-assisted laser desorption/ionization time-of-flight mass spectroscopy (MALDI-ToF/MS, Bruker, Bruker autoflex Ⅲ) was also used to identify DACSM. POM images at different temperatures were obtained by a cross-polarized optical microscope (POM, Nikon, ECLIPSE E600POL) with a microscope hot stage system (Mettler Toledo, HS82). Thermal phase transition behavior of DACSM was monitored using differential scanning calorimetry (DSC, Perkin Elmer, DSC 4000). To understand the

molecular packing structure of DACSM, a wide-angle X-ray diffraction instrument (WAXD, Bruker, D8 ADVANCE) was used. The positions of diffraction peaks were calibrated with a silicon crystal (2θ = 28.466). The absorption (or transmittance) and emission properties of differently treated DACSM samples were investigated by UV-Vis spectrometer (Scinco, S-3100) and spectro-fluorophotometer (Shimadzu, RF-6000). The formation of polydiacetylene under 254 nm light and its conformational change was confirmed by Raman spectroscopy (Nanophoton, RAMAN Touch). Photoluminescence quantum yield and lifetime measurements were conducted using a spectro-fluorophotometer (Horiba, Fluorolog-3 with TCSPC).

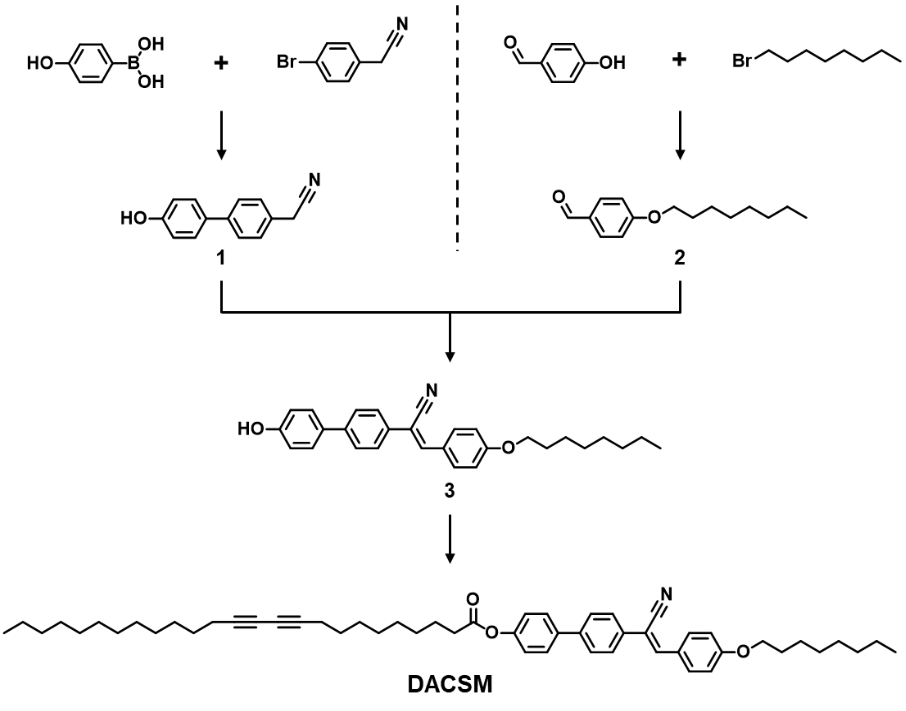

Figure S1. Synthetic procedure of DACSM.

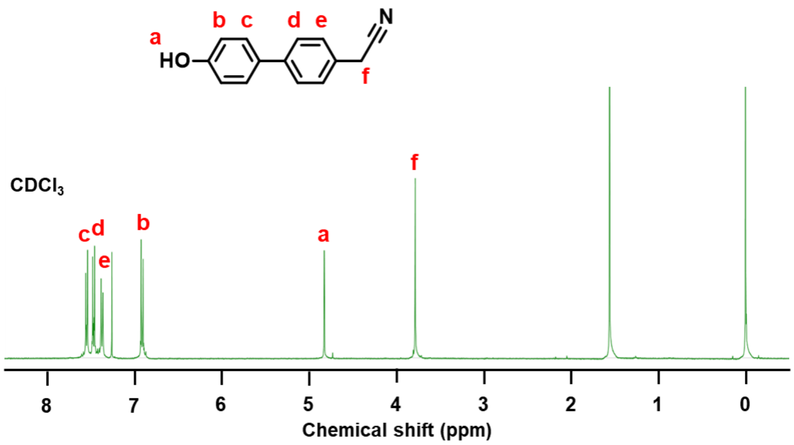

Figure S2. ^{1}H NMR spectrum of Compound 1.

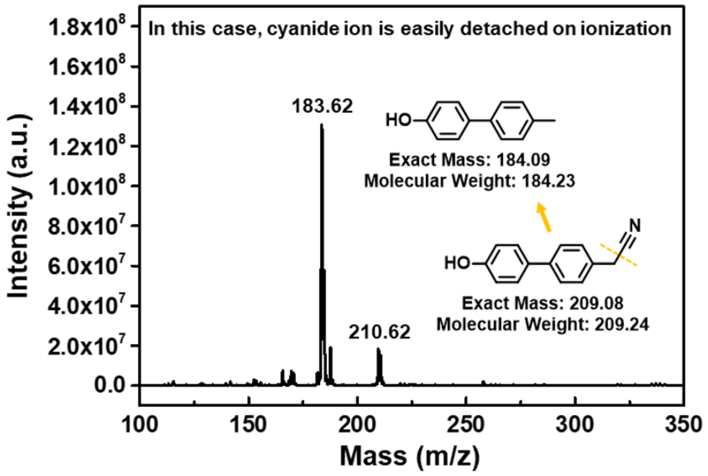

Figure S3. GC/MS spectrum of Compound 1.

.

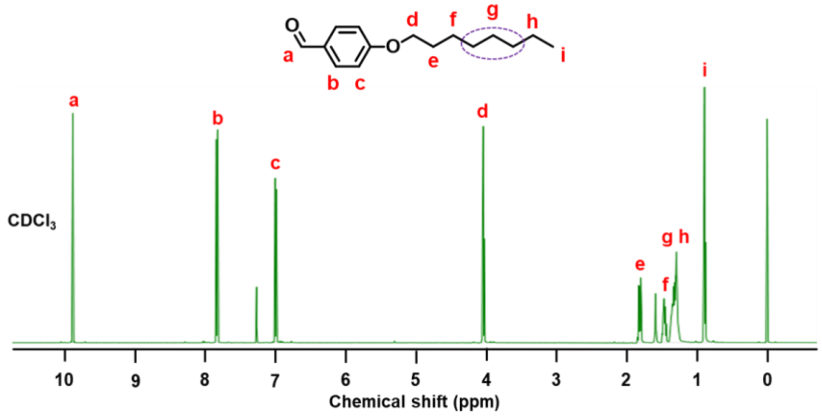

Figure S4. ^{1}H NMR spectrum of Compound 2.

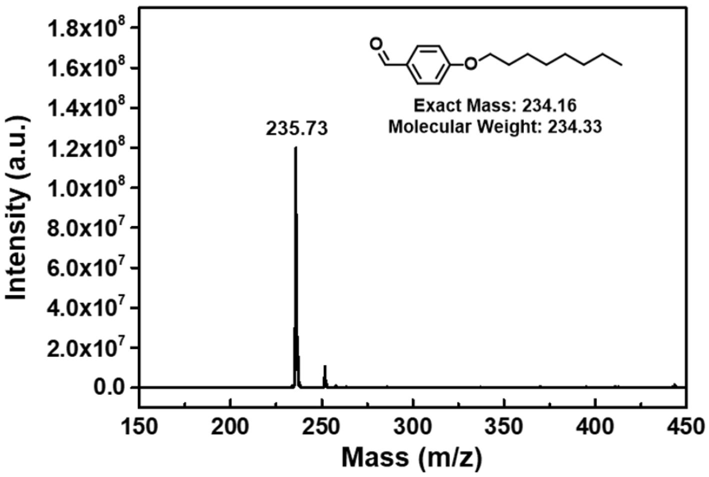

Figure S5. GC/MS spectrum of Compound 2.

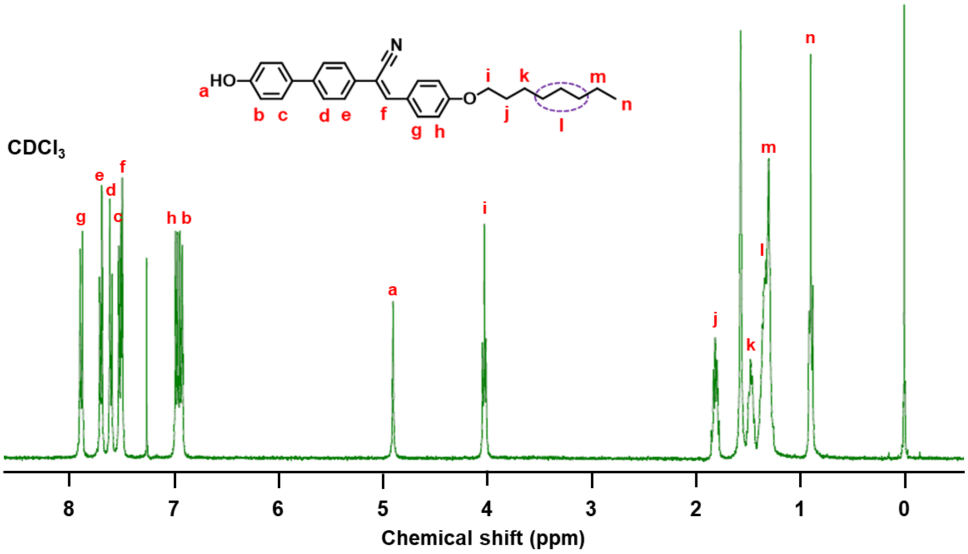

Figure S6. ^{1}H NMR spectrum of Compound 3.

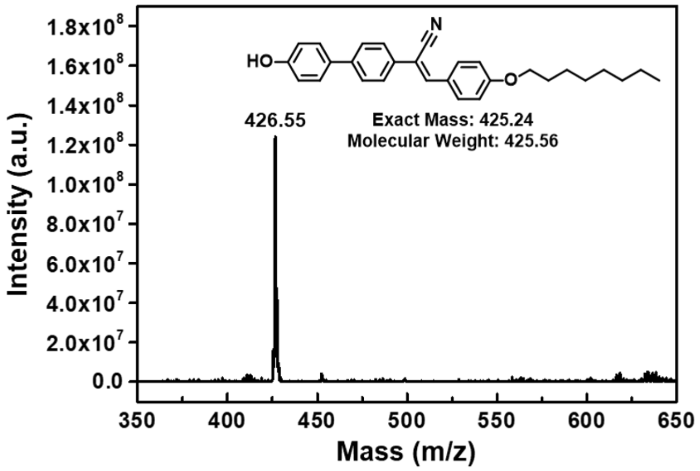

Figure S7. GC/MS spectrum of Compound 3.

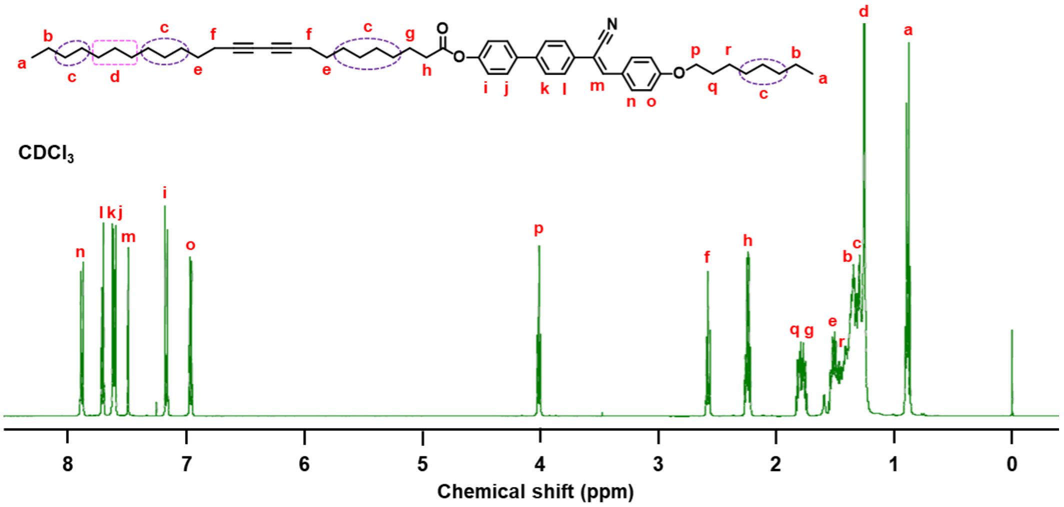

Figure S8. ^{1}H NMR spectrum of DACSM.

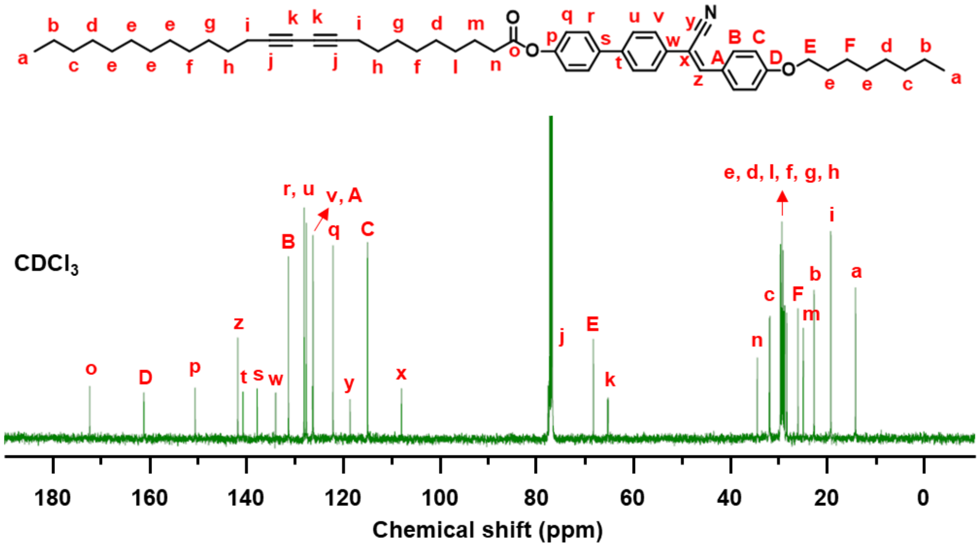

Figure S9. ^{13}C NMR spectrum of DACSM.

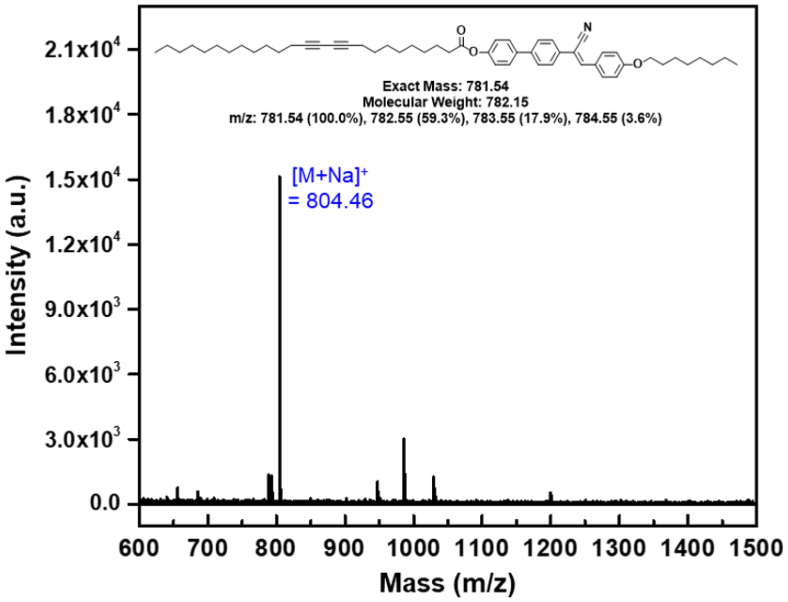

Figure S10. MALDI-ToF MS data of DACSM.

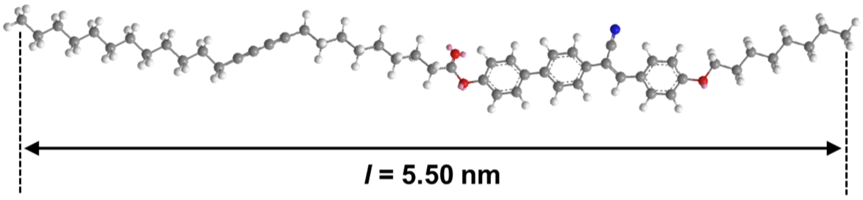

Figure S11. The optimized molecular geometry of DACSM.

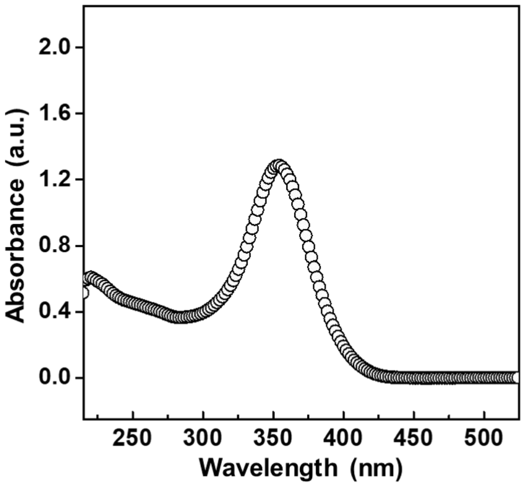

Figure S12. UV-Vis spectrum of DACSM in THF (concentration: 60 μM).

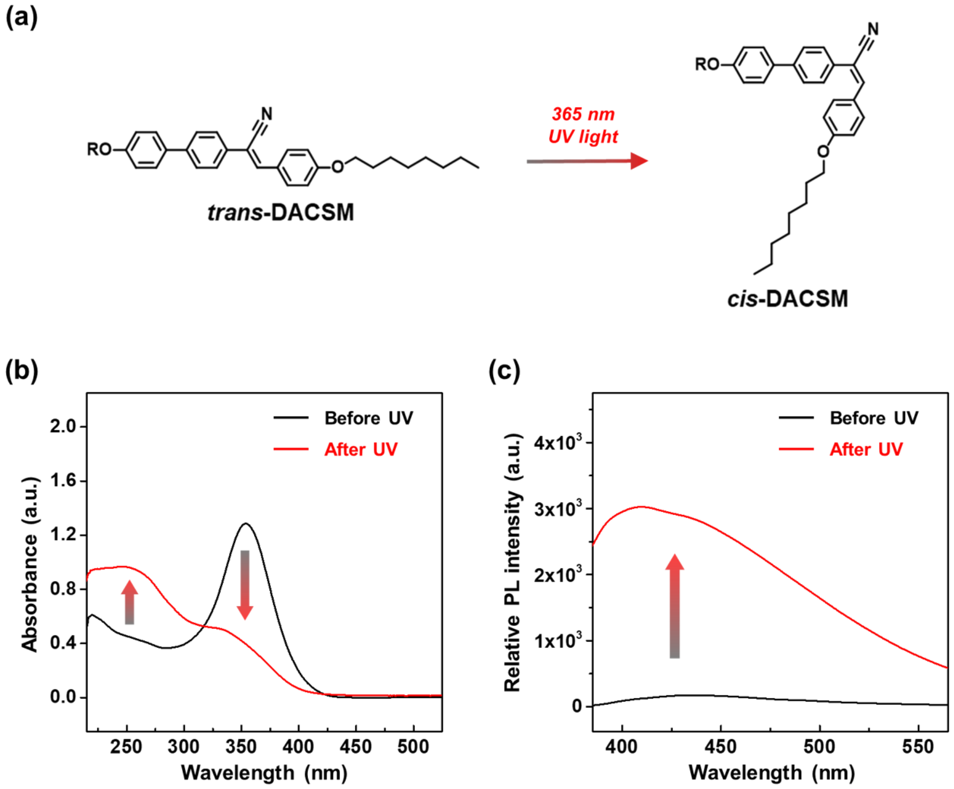

Figure S13. a) Photoisomerization of DACSM from *trans*- to *cis* form. b) UV-Vis and c) PL spectra of DACSM in THF before and after UV irradiation.

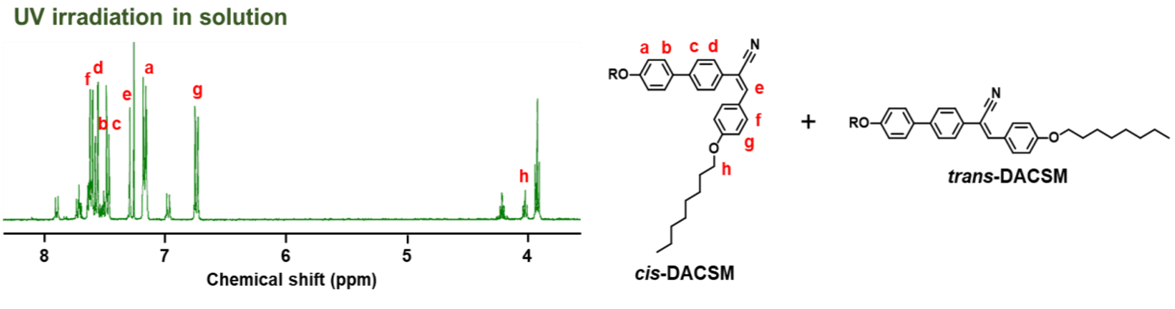

Figure S14. ^{1}H NMR spectrum of DACSM in $CDCl_3$ after UV irradiation.

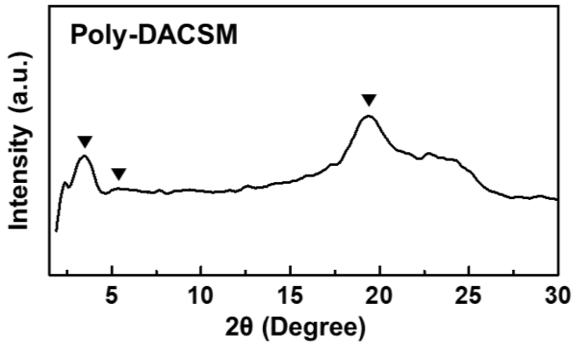

Figure S15. 1D WAXD pattern of Poly-DACSM.

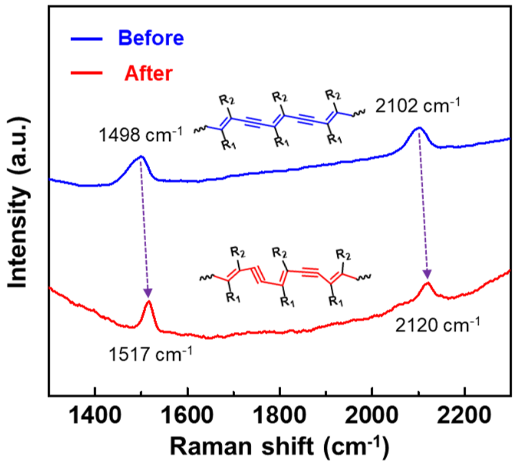

Figure S16. Raman spectra of Poly-DACSM upon heat treatment.

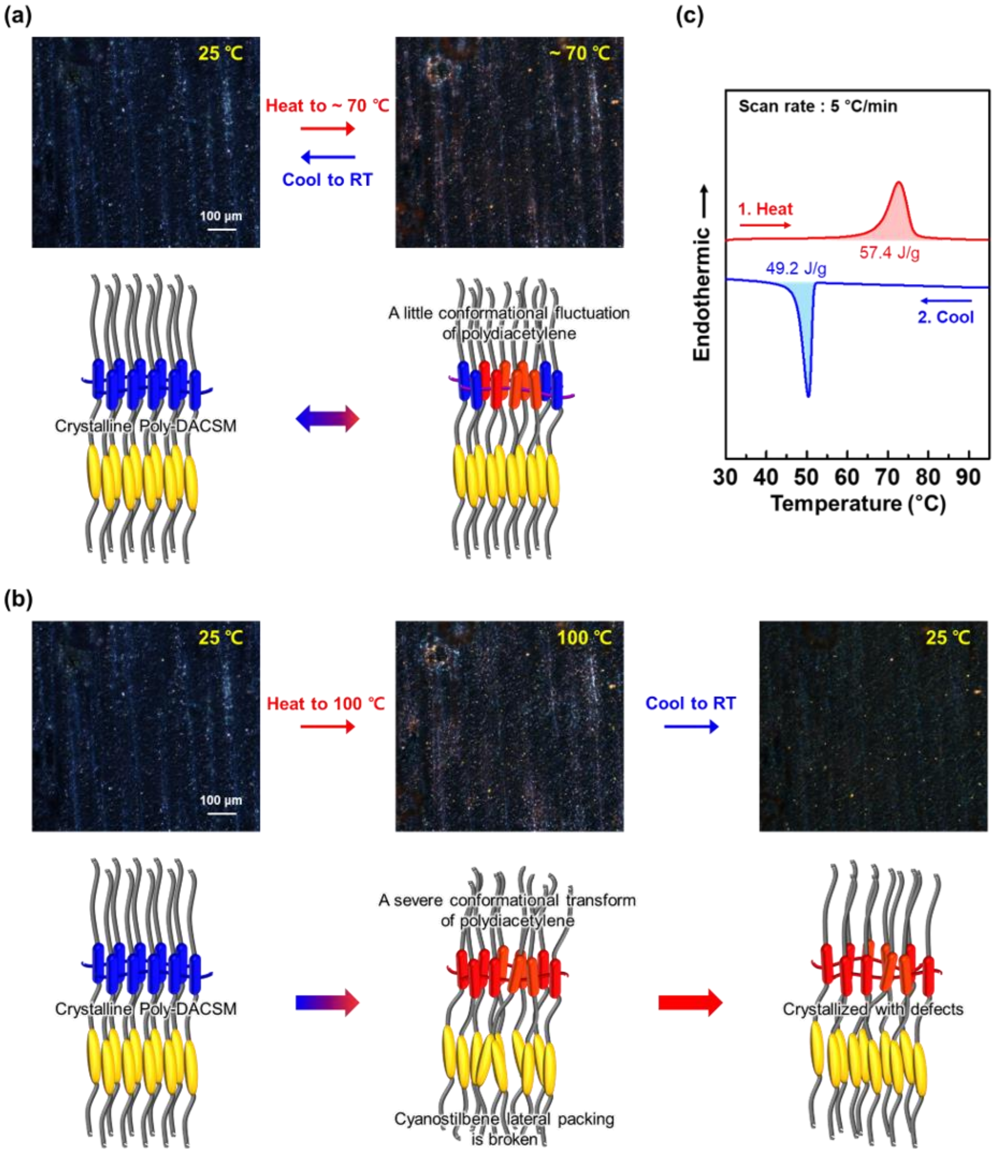

Figure S17. POM textures of Poly-DACSM a) within 70 °C, and b) upon heating to 100 °C and subsequent cooling to 25 °C. c) DSC trace of Poly-DACSM between 30 °C and 100 °C.

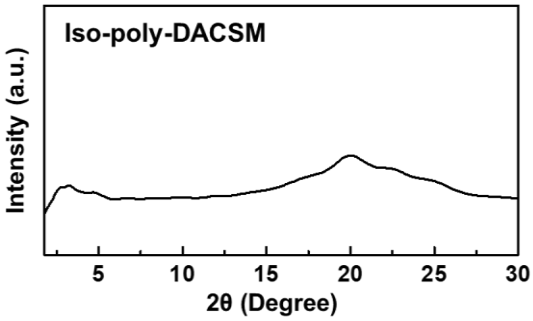

Figure S18. 1D WAXD pattern of Iso-poly-DACSM.

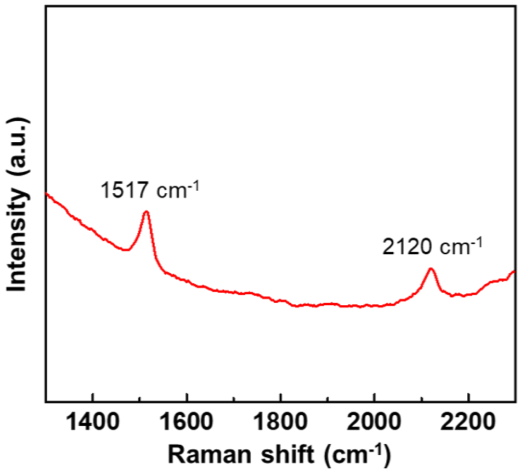

Figure S19. Raman spectrum of Iso-poly-DACSM.

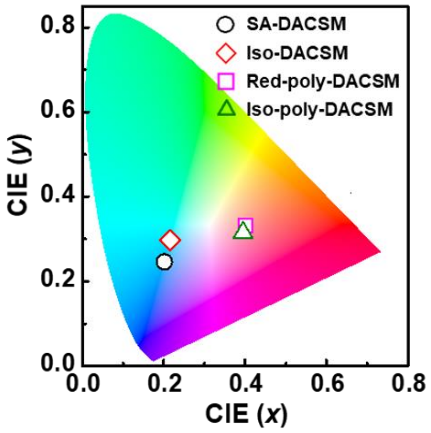

Figure S20. CIE diagram for PL color of different moleulcar states.

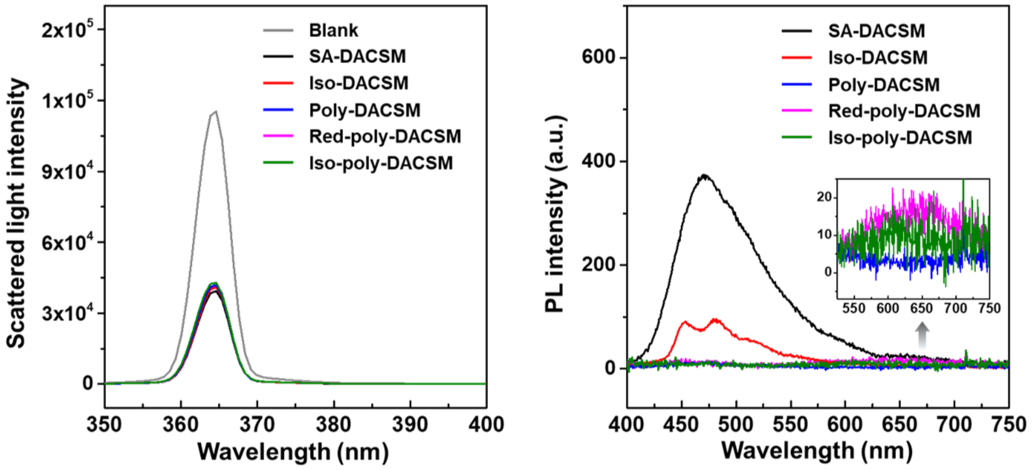

Samples	L_n	L_{Blank}	E_n - E_{Blank}	QY (%)
SA-DACSM	218032	604119	38954	10.1
Iso-DACSM	226727	604119	9049	2.4
Poly-DACSM	233038	604119	1197	0.3
Red-poly-DACSM	228224	604119	4196	1.1
Iso-poly-DACSM	241124	604119	3251	0.9

Figure S21. PL quantum yield results of five different DACSM states.

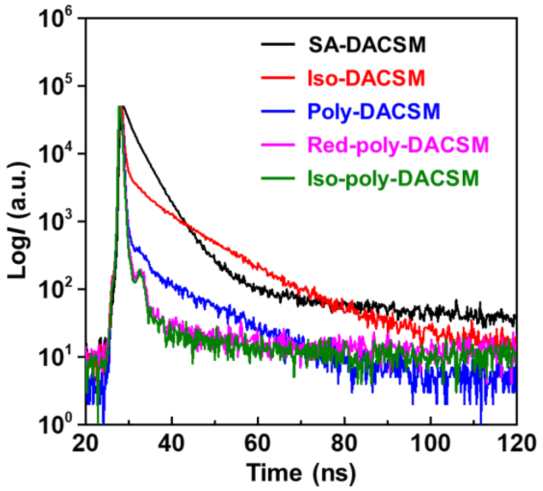

Figure S22. PL lifetime decay of five different DACSM states.

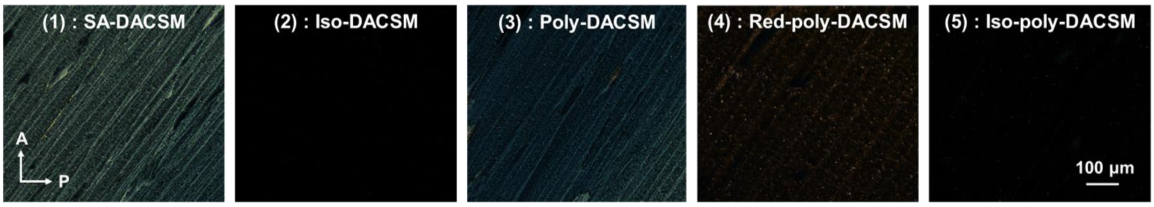

Figure S23. POM textures of five different DACSM molecular states in (1) - (5) areas.
